# COVID-19 detection and heatmap generation in chest x-ray images

**DOI:** 10.1117/1.JMI.8.S1.014001

**Published:** 2021-01-09

**Authors:** Worapan Kusakunniran, Sarattha Karnjanapreechakorn, Thanongchai Siriapisith, Punyanuch Borwarnginn, Krittanat Sutassananon, Trongtum Tongdee, Pairash Saiviroonporn

**Affiliations:** a Mahidol University, Faculty of Information and Communication Technology, Nakhon Pathom, Thailand; b Mahidol University, Department of Radiology, Siriraj Hospital, Bangkok, Thailand

**Keywords:** COVID-19, chest x-ray, heatmap, lung detection, ResNet

## Abstract

**Purpose:** The outbreak of COVID-19 or coronavirus was first reported in 2019. It has widely and rapidly spread around the world. The detection of COVID-19 cases is one of the important factors to stop the epidemic, because the infected individuals must be quarantined. One reliable way to detect COVID-19 cases is using chest x-ray images, where signals of the infection are located in lung areas. We propose a solution to automatically classify COVID-19 cases in chest x-ray images.

**Approach:** The ResNet-101 architecture is adopted as the main network with more than 44 millions parameters. The whole net is trained using the large size of 1500×1500 x-ray images. The heatmap under the region of interest of segmented lung is constructed to visualize and emphasize signals of COVID-19 in each input x-ray image. Lungs are segmented using the pretrained U-Net. The confidence score of being COVID-19 is also calculated for each classification result.

**Results:** The proposed solution is evaluated based on COVID-19 and normal cases. It is also tested on unseen classes to validate a regularization of the constructed model. They include other normal cases where chest x-ray images are normal without any disease but with some small remarks, and other abnormal cases where chest x-ray images are abnormal with some other diseases containing remarks similar to COVID-19. The proposed method can achieve the sensitivity, specificity, and accuracy of 97%, 98%, and 98%, respectively.

**Conclusions:** It can be concluded that the proposed solution can detect COVID-19 in a chest x-ray image. The heatmap and confidence score of the detection are also demonstrated, such that users or human experts can use them for a final diagnosis in practical usages.

## 1 Introduction

Chest x-ray images could be used in an automatic image analysis for detecting and classifying abnormalities, including diseases of atelectasis, consolidation, infiltration, pneumothorax, edema, emphysema, fibrosis, effusion, pneumonia, pleural thickening, cardiomegaly, nodule mass, and hernia, as mentioned in the NIH chest x-ray dataset.^1^ On top of these diseases, which are commonly diagnosed using chest x-ray images, this paper focuses on the new disease of COVID-19 which causes lung damage and can also be detected in chest x-ray images.^2^ Several methods were proposed recently to address this research domain. Existing methods are reviewed below. All of them were developed based on the convolutional neural network (CNN).

Zhang et al.^3^ adopted 18 layers of a residual CNN that was pretrained with ImageNet dataset. The sigmoid was used as an activation function, with the binary cross-entropy loss function. Differently, Wang et al.^4^ trained the model to classify a chest x-ray image into three classes including normal, COVID-19, and other viral and bacterial diseases. Its model was constructed using five convolutional layers of first-stage projection, expansion, depthwise representation, second-stage projection, and extension. Narin et al.^5^ attempted using three different types of CNN architectures, including ResNet50, InceptionV3, and Inception-ResNetV2. It was found that the pretrained ResNet50 using ImageNet provided the highest classification performance. While, Apostolopoulos and Mpesiana^6^ attempted using five different types of CNN architectures, including VGG19, MobileNet v2, Inception, Xception, and Inception ResNet v2. However, their research reported the best performance on using MobileNet v2 with the transfer learning of ImageNet.

Hemdan et al.^7^ tried seven different types of CNN architectures, including VGG19, DenseNet121, InceptionV3, ResNetV2, Inception-ResNet-V2, Xception, and MobileNetV2. They recommended two models, VGG19 and DenseNet201, which achieved the best classification accuracy. Afshar et al.^8^ proposed a CNN architecture containing four convolutional layers and three capsule layers, by taking three-dimensional x-ray images as the input. The model could classify four labels of normal, bacterial, non-COVID viral, and COVID-19. Abbas et al.^9^ used a CNN model of DeTraC transformation pretrained using ImageNet as the backbone architecture. Principal component analysis was used to reduce the dimension of the extracted feature, and the nearest centroid based on the squared Euclidean distance was applied as the main classification. Li et al.^10^ attempted using three lightweight CNN architectures of MobileNetV2, ShuffleNetV2, and SqueezeNet. Karim et al.^11^ reported the best performance by combining VGG-19 and DenseNet-161.

Similarly, Minaee et al.^12^ trained their model by trying four CNN architectures, including ResNet18, ResNet50, SqueezeNet, and DenseNet-121. The SqueezeNet provided the best performance. Mangal et al.^13^ used the CheXNet based on 121-layer DenseNet which was trained on ChestX-ray14.^14^ In addition, Khan et al.^15^ also developed a COVID-19 classification model using CNN with the inception pretrained based on ImageNet dataset. Similarly, Hall et al.^16^ and Bukhari et al.^17^ both adopted Resnet50 pretrained using ImageNet dataset.

As can be seen from existing methods in the literature, they were developed using CNN-based approaches with various well-known architectures. Their results are reported and compared in Sec. 3. They used pretrained networks that were trained on either the popular ImageNet dataset or the related chest x-ray images dataset, such as ChestX-ray14. Therefore, they had to limit the size of input images to be the same size as that used in the pretrained models, which was usually small. Another important reason for using a small size of input image is a limited computational resource of RAM and GPU. This may not be able to cope with a small-sized signal of COVID-19 in a chest x-ray image.

This paper proposes a solution using ResNet-101, 101 layers deep.^18^ Although it has a version with pretrained weights on ImageNet database,^19^ the proposed method adopts ResNet-101 as a backbone architecture but is trained from scratch. This is mainly because the pretrained model was learned on a small size input image of 224×224  pixels, which may fail to detect some small signals of the COVID-19 in a chest x-ray image. This paper develops a solution model by taking a large-sized input image of 1500×1500  pixels.

Importantly, in our experiments, the proposed method is evaluated using four subdatasets of D1: COVID-19, D2: normal (without any diseases and remarks), D3: other normal (without any diseases but with some remarks), and D4: other abnormal (with other diseases). Our models are trained for two versions of (1) classifying into two classes of COVID-19 and non-COVID-19 and (2) classifying into three classes of COVID-19, normal, and other normal together with other abnormal.

In this paper, the experimental results are reported in terms of confusion matrix, accuracy, sensitivity, and specificity, under different cut-off thresholds of the confidence value in the output layer of the model. Heatmaps emphasizing signals of COVID-19 in chest x-ray images are computed from filtering kernels of the model. They are visualized in the region of segmented lungs using U-Net, since COVID-19 is supposed to be within the lung region in chest x-ray images.

The rest of this paper is organized as follows. Section 2 explains the proposed method of classifying COVID-19 in chest x-ray images. Section 3 illustrates the experimental results in various scenarios. Then, results are discussed in Sec. 4, and conclusions are summarized in Sec. 5.

## 2 Materials and Methods

**Fig. 1:**
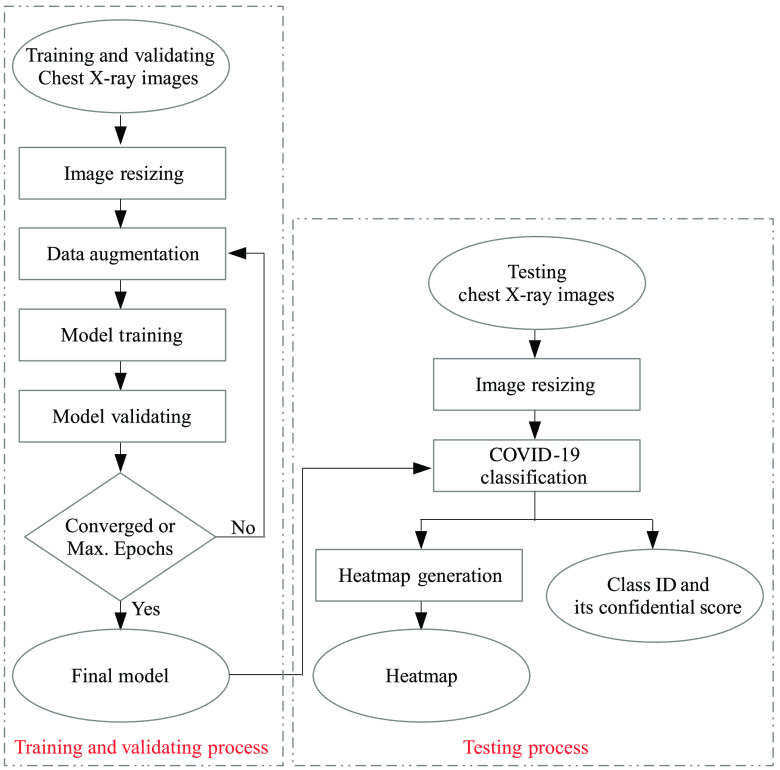
Overview framework of the proposed method.

Figure 1 shows an overview framework of the proposed solution. The training, validating, and testing chest x-ray images are resized into 1500×1500  pixels.^20^ Then, the real-time data augmentation is applied on the original training images. Both original and augmented training images are fed to train the classification model based on the backbone architecture as explained below. The trained model is then validated with the original validating images. If the validating result is converged or the maximum number of epochs is reached, then the training and validating processes are stopped and the final model is concluded. Otherwise, it goes back to the data augmentation process and repeats to the next epoch.

In the testing phase, the trained model is applied on each resized chest x-ray image, to compute predicted class, its confidence score, and heatmap. The details are explained in the following sections.

### 2.1 Backbone Architecture

The ResNet-101^21^^,^^22^ is adopted in our proposed method as the backbone architecture of the COVID-19 classification model. It is a very deep network containing deep layers as shown in Table 1,^23^ with more than 44 millions parameters.

**Table 1:** The convolutional layers in ResNet-101 used in the proposed solution.

Layer name	Building block of (kernel size, filter)	Number of building blocks
conv1	[7×7,64]	1
conv2	$\left[\begin{array}{c} 1\times 1,64\\ 3\times 3,64\\ 1\times 1,256 \end{array}\right]$	3
conv3	$\left[\begin{array}{c} 1\times 1,128\\ 3\times 3,128\\ 1\times 1,512 \end{array}\right]$	4
conv4	$\left[\begin{array}{c} 1\times 1,256\\ 3\times 3,256\\ 1\times 1,1024 \end{array}\right]$	23
conv5	$\left[\begin{array}{c} 1\times 1,512\\ 3\times 3,512\\ 1\times 1,2048 \end{array}\right]$	3

**Fig. 2:**
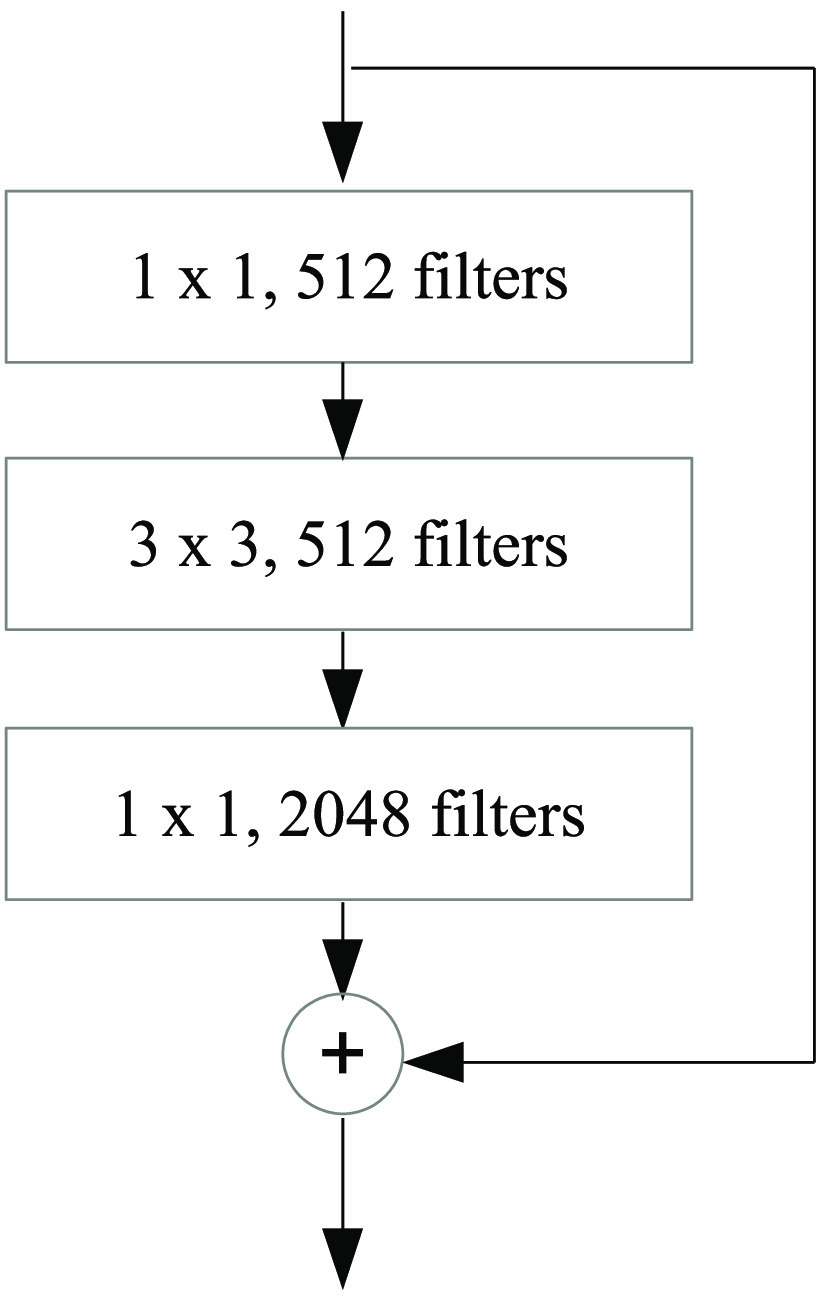
A building block in conv5 of ResNet-101.

As shown in Table 1, each square bracket represents each building block of convolutional layers which are parametrized by kernel size and filter. For example, the kernel size of 7×7 means the height and width of the two-dimensional convolution window are both 7. While, the filter refers to the dimensionality of the output space or the number of output filters in each convolutional layer.^24^ The third column represents a number of defined building blocks used in each convolutional layer. For example, in the layer named “conv5,” each building block contains three convolutional layers connected in sequence as shown in Fig. 2.

The input to the network is a chest x-ray image, as shown in Fig. 1. The ResNet-101 has a version with pretrained weights using ImageNet dataset which is a large-scale classification dataset containing 1.2 million training images from 1000 classes of objects.^22^ However, the input image’s size must be limited to the pretrained requirement of 224×224  pixels. This may not cope well in the case of differentiating normal class from other-normal classes.

In this paper, the ResNet-101 is trained from scratch using the input images of the large size 1500×1500  pixels. The top part (i.e., classification part) of ResNet-101 is replaced with the global average pooling, softmax, and output layers. Five types of data augmentations are added on the training dataset, including zoom, rotate, shear, flip, and shift.^25^

The proposed solution develops two types of models which are different in the output layer. The first model is developed to classify COVID-19 class from any non-COVID-19 class, having two nodes in the output layer. While, the second model is developed to classify a chest x-ray image into three classes having three nodes in the output layer of COVID-19, normal without any diseases, and norther normal with some other diseases or remarks.

### 2.2 Lung Segmentation

In this paper, the lung segmentation is required in the step of heatmap visualization, where the color maps are shown in the area of segmented lungs only. The pretrained U-Net-based model^26^ is adopted in the proposed solution of lung segmentation, since it has been successfully used for the medical image segmentation. The U-Net contains two main activities of convolution and transposed convolution. The transposed convolution is a process to increase the spatial resolution of the input by upsampling the kernel.

It is called U-Net because its architecture looks like a U shape, where a front side of the U-shape contains convolution layers for downsampling and a back side of the U-shape contains transposed convolution layers for upsampling. The convolution and transposed convolution layers of the U-shape are summarized in Table 2.

**Table 2:** The convolution and transposed convolution layers of the U-shape used in the proposed solution.

Front side of the U-shape	Back side of the U-shape
$\left[\begin{array}{c} 3\times 3,64\\ 3\times 3,64 \end{array}\right]$	$\left[\begin{array}{c} 3\times 3,64\\ 3\times 3,64 \end{array}\right]$
↓ (max pool 2×2)	↑ (up-conv 2×2)
$\left[\begin{array}{c} 3\times 3,128\\ 3\times 3,128 \end{array}\right]$	$\left[\begin{array}{c} 3\times 3,128\\ 3\times 3,128 \end{array}\right]$
↓ (max pool 2×2)	↑ (up-conv 2×2)
$\left[\begin{array}{c} 3\times 3,256\\ 3\times 3,256 \end{array}\right]$	$\left[\begin{array}{c} 3\times 3,256\\ 3\times 3,256 \end{array}\right]$
↓ (max pool 2×2)	↑ (up-conv 2×2)
$\left[\begin{array}{c} 3\times 3,512\\ 3\times 3,512 \end{array}\right]$	$\left[\begin{array}{c} 3\times 3,512\\ 3\times 3,512 \end{array}\right]$
↓ (max pool 2×2)	↑ (up-conv 2×2)
$\left[\begin{array}{c} 3\times 3,1024\\ 3\times 3,1024 \end{array}\right]$	

The input layer is connected to the first building block of the front side of U-shape. While, the output layer of two nodes (i.e., lung and non-lung nodes) is connected to the last building block of the back side of U-shape. The pretrained U-Net is adopted for the lung segmentation.^27^ It reported the Dice similarity coefficients of 0.985 and 0.972 on the datasets of Montgomery and JSRT,^28^ respectively. In addition, the average size of segmented lungs is about 29.8% of the original size of input images.

### 2.3 Heatmap Generation

As shown in Fig. 1, the heatmap is generated for each test x-ray image. Since there are many layers and a large number of filters, the average of the filters’ weights of the last convolutional layer is calculated and visualized. This is because they could represent the feature maps directly. The key steps are listed below.

• A test chest x-ray image is fed into the trained ResNet-101 model. The predicted filters’ weights are also computed at this stage.
• All the filters’ weights in the last convolutional layer are extracted.
• The average weight from all filters’ weights is calculated.
• The average weight is used as a mask on the test chest x-ray image to generate the heatmap.
• The heatmap is visualized only on the lung areas segmented by the pretrained U-Net.

## 3 Results

This section explains and discusses our experimental results on different scenarios. Both our own dataset and published dataset^4^ are used in the experiments, as shown in Table 3. For the published dataset, only chest x-ray images with COVID-19 are used in our experiments, because they are used to validate the cross-datasets scenario of COVID-19 detection.

**Table 3:** Datasets used in the experiments to validate the proposed solution.

Dataset symbol	Description	Number of images	Reference
D1	COVID-19	142	Newly collected dataset
D2	Normal without any diseases or remarks	5218	Newly collected dataset
D3	Other normal in elderly patients with minimal fibrosis and spondylosis of spine	100	Newly collected dataset
D4	Other abnormal including tuberculosis, pneumonia, and pulmonary edema	100	Newly collected dataset
D5	Taking only chest x-ray images of COVID-19 from Ref. 4	183	Wang et al.^4^

In addition, in our D1 dataset, the 142 images of COVID-19 cases were obtained from three levels of the severity as: (1) 22 images of the severe level, (2) 13 images of the moderate level, and (3) 107 images of the mild level. Each patient case has only one image taken in each instance. In the training and testing processes, each individual image is fed as the input into the CNN-based model at a time.

Five datasets, as listed in Table 3, are used in our four scenarios of experiments as below. The results are reported in terms of confusion matrix, accuracy, sensitivity, and specificity.

• Scenario 1. Two classes prediction: COVID-19 (class 1) and non-COVID-19 (class 2); train and validate: 100 images from D1 (class 1) and 100 images from D2 (class 2); test: 42 images from D1 (class 1), 5118 images from D2 (class 2), 100 images from D3 (class 2), 100 images from D4 (class 2).
• Scenario 2. Two classes prediction: COVID-19 (class 1) and non-COVID-19 (class 2); train and validate: 100 images from D1 (class 1), 40 images from D2 (class 2), 30 images from D3 (class 2), 30 images from D4 (class 2); test: 42 images from D1 (class 1), 5178 images from D2 (class 2), 70 images from D3 (class 2), 70 images from D4 (class 2).
• Scenario 3. Two classes prediction: COVID-19 (class 1) and non-COVID-19 (class 2); train and validate: 100 images from D1 (class 1) and 100 images from D2 (class 2); test: 183 images from D5 (class 1).
• Scenario 4. Three classes prediction: COVID-19 (class 1), normal (class 2), and other normal (class 3); train and validate: 100 images from D1 (class 1), 100 images from D2 (class 2), 50 images from D3 (class 3), and 50 images from D4 (class 3); test: 42 images from D1 (class 1), 5118 images from D2 (class 2), 50 images from D3 (class 3), 50 images from D4 (class 3).

Images from the five datasets (D1 to D5) are independently split into two subsets of (1) training and validating set and (2) testing set, as mentioned in each scenario. Later, the training and validating set is further randomly split into the training set and validating set with proportions of 90% and 10%, respectively, in each epoch of the CNN training phase. So, in all cases, images in training, validating, and testing sets are independent and nonoverlapped. The numbers of independent images in training, validation, and testing arrangements of each scenario are summarized in Table 4.

**Table 4:** The numbers of independent images from the five datasets (D1 to D5) used in training, validation, and testing arrangements of each scenario.

Scenario	Train					Validate					Test				
	D1	D2	D3	D4	D5	D1	D2	D3	D4	D5	D1	D2	D3	D4	D5
1	90	90	—	—	—	10	10	—	—	—	42	5118	100	100	—
2	90	36	27	27	—	10	4	3	3	—	42	5178	70	70	—
3	90	90	—	—	—	10	10	—	—	—	—	—	—	—	183
4	90	90	45	45	—	10	10	5	5	—	42	5118	50	50	—

In this paper, the positive class is drawn when the confidence value predicted by the trained CNN-based model is higher than the cut-off score. The descriptive statistical analysis is used to determine the results in terms of sensitively, specificity, and accuracy. These performances are also compared with other existing methods in the literature.

### 3.1 Scenario 1

This scenario is designed to validate the constructed model on two classes of COVID-19 and non-COVID-19. It is trained, validated, and tested on chest x-ray images of COVID-19 cases and normal cases without any diseases or remarks. Also, it is tested on unseen/untrained datasets of D3 and D4, which have other diseases or remarks similar to COVID-19. The confusion matrix is shown in Table 5.

**Table 5:** Experimental results of scenario 1.

True labels	Predicted classes	
	COVID-19 (class 1)	Non-COVID-19 (class 2)
COVID-19 (class 1, D1)	97%	3%
Non-COVID-19 (class 2, D2)	2%	98%
Non-COVID-19 (class 2, D3+D4)	85%	15%

As shown in Table 5, considering only trained datasets (i.e., D1 and D2), the sensitivity and specificity are 97% and 98%, respectively. However, if taking both seen and unseen datasets into consideration (i.e., D1, D2, D3, and D4), the specificity is dropped, especially the unseen datasets of D3 and D4. Rather than using the predictions directly from the output layer (as shown in Table 5), the predictions of COVID-19 are calculated using the cut-off of 90% confidence scores. Its confusion matrix is shown in Table 6.

**Table 6:** Experimental results of scenario 1, using the cut-off of 90% confidence scores.

True labels	Predicted classes	
	COVID-19 (class 1)	Non-COVID-19 (class 2)
COVID-19 (class 1, D1)	83%	17%
Non-COVID-19 (class 2, D2)	0%	100%
Non-COVID-19 (class 2, D3+ D4)	56%	44%

Since the cut-off score of COVID-19 is increased, the specificity is also increased on both seen and unseen datasets. However, the specificity on unseen datasets is still not promising. Another side-effect is that the sensitivity is getting lower. It is not sensible to lower the sensitivity for the medical diagnosis. Even the unseen datasets contain non-COVID-19 images, but they could be confused with COVID-19 class because they contain diseases or remarks on lungs similar to COVID-19 cases. Therefore, in the scenario 2, the datasets D3 and D4 will be included in the non-COVID-19 class for training.

### 3.2 Scenario 2

The scenario 2 is designed to extend the scenario 1 by adding the datasets D3 and D4 into the training process. So, the model could also learn the non-COVID-19 cases that have remarks on lungs of other diseases. The confusion matrix is shown in Table 7. The specificity on the datasets D3 and D4 is now significantly higher, when compared with the result shown in the scenario 1. This is because they are now also used in the learning process. However, the sensitivity is lower, when compared with the result in the scenario 1. It could be because the COVID-19 images are confused with the images of other diseases. This can be solved by splitting the problem into three classes instead of two classes, which will be discussed in the scenario 4.

**Table 7:** Experimental results of scenario 2.

True labels	Predicted classes	
	COVID-19 (class 1)	Non-COVID-19 (class 2)
COVID-19 (class1, D1)	73%	27%
Non-COVID-19 (class 2, D3 +D4)	7%	93%

The additional experiments are conducted in this scenario, to see the tradeoff between the accuracy and the training time when increasing the size of input images. The results are reported as: (1) using 1500×1500  pixels, the accuracy of predicting COVID-19 class is 73%, the accuracy of predicting non-COVID-19 class is 93%, and the training time is 2 h and 27 min; (2) using 1000×1000  pixels, the accuracy of predicting COVID-19 class is 40%, the accuracy of predicting non-COVID-19 class is 100%, and the training time is 1 h and 13 min; (3) using 500×500  pixels, the accuracy of predicting COVID-19 class is 0%, the accuracy of predicting non-COVID-19 class is 100%, and the training time is 34 min. The models are trained using NVIDIA-V100 Tensor Core. However, the testing time is very fast and not significantly different among these three different sizes of input images. Therefore, in this paper, the size of 1500×1500  pixels is used as it is the maximum size in which our machine’s memory can handle in the training process.

### 3.3 Scenario 3

The scenario 3 is designed to test the trained model from the scenario 1, with the COVID chest x-ray images of unseen dataset (i.e., D5). The classification results are calculated based on two different cut-off values of 50% and 90% on the confidence scores, as shown in Table 8.

**Table 8:** Experimental results of scenario 3 on chest x-ray images of COVID-19, using the cut-off of 50% or 90% confidence scores.

Cut-off value	Predicted classes	
50%	93%	7%
90%	84%	16%

The proposed solution could achieve the high sensitivity score of 93% on the cross-dataset scenarios, where D1 and D2 are used for training and validating, but unseen D5 is used for testing. This shows the regularization of the constructed model of COVID-19 classification.

### 3.4 Scenario 4

The scenario 4 is designed for the experiment of classifying chest x-ray images into three classes including COVID-19 (class 1), normal (class 2), and other normal (class 3). The confusion matrix is shown in Table 9.

**Table 9:** Experimental results of scenario 4.

True labels	Predicted classes		
	COVID-19 (class 1)	Normal (class 2)	Other normal (class 3)
COVID-19 (class 1, D1)	80%	0%	20%
Normal (class 2, D2)	0%	83%	17%
Other normal (class 3, D3 + D4)	3%	0%	97 %

As shown in Table 9, the class 1 of COVID-19 and the class 3 of other normal are confused with each other in some extent. This is because the class 3 contains chest x-ray images having remarks similar to COVID-19. They were recorded from elderly patients with minimal fibrosis and spondylosis of spine, and patients with other diseases including tuberculosis, pneumonia, and pulmonary edema. In addition, the class 2 is confused with the class 3 because they share the common features of non-COVID-19.

### 3.5 Comparisons

Table 10 shows the experimental results of the proposed method and other existing methods in the literature. This is considered to be the indirect comparison since they are tested on different datasets. The performances of all methods are comparable. However, the proposed method achieves the best average score (97.7%) of three values of sensitivity, specificity, and accuracy.

**Table 10:** Experimental comparisons.

Method	Dataset	Sensitivity (%)	Specificity (%)	Accuracy (%)
Zhang et al.^3^	100 images of COVID-19, 1431 images of pneumonia	96	71	95
Wang et al.^4^	183 images of COVID-19, 8066 patient cases with no pneumonia, 5538 patient cases with non-COVID19 pneumonia	87	99	93
Narin et al.^5^	50 images of COVID-19, 50 images of normal	100	—	98
Apostolopoulos and Mpesiana^6^	224 images of COVID-19, 700 images of common bacterial pneumonia, 504 images of normal	99	97	93
Hemdan et al.^7^	25 images of COVID-19, 25 images of normal	—	—	90
Abbas et al.^9^	105 images of COVID-19, 80 images of normal, 11 images of SARS	98	92	95
Khan et al.^15^	284 images of COVID-19, 310 images of normal, 330 images of pneumonia bacterial, 327 images of pneumonia viral	—	—	90
Hall et al.^16^	135 images of COVID-19, 320 images of viral and bacterial pneumonia	83	98	94
Minaee et al.^12^	40 images of COVID-19, 3000 images of normal	97	98	—
Mangal et al.^13^	165 images of COVID-19, 1583 images of normal	100	—	91
Bukhari et al.^17^	89 images of COVID-19, 93 images of lungs without any radiological abnormality, 96 images with pneumonia caused by other pathogens	—	—	98
Proposed method	See Table 3	97	98	98

Using a large size of input images in this paper, our proposed method could achieve a better performance when compared with using a smaller size of input images. This is because of two main reasons. First, signals of COVID-19 in each image contain a larger number of pixels. This is useful in the training process especially when the proportion of COVID-19’s signals is small. Second, the distortion from reducing size of the original image appears to be less because the reduction ratio is smaller.

### 3.6 Heatmaps and Confidence Scores

**Fig. 3:**
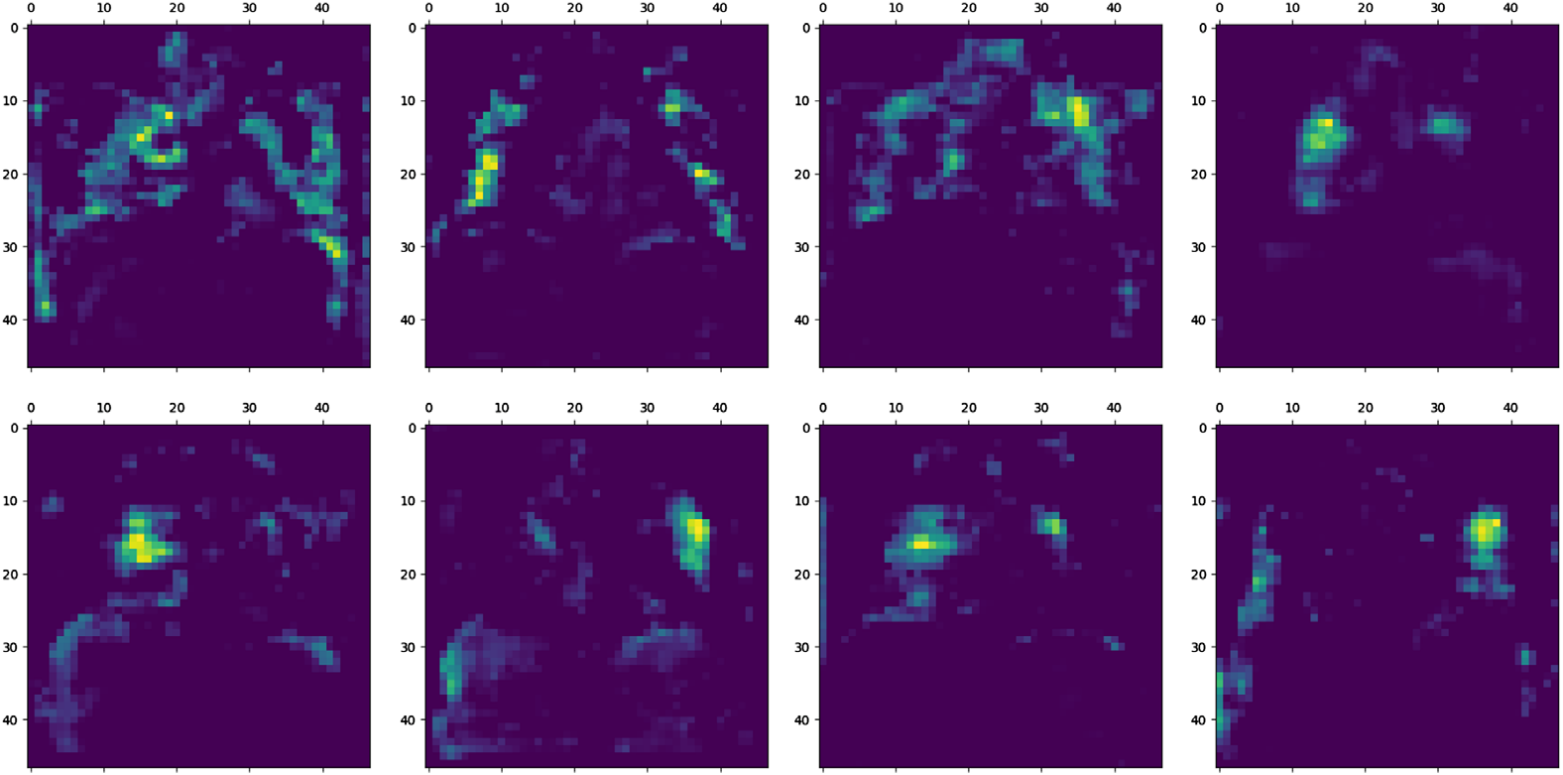
Sample filters’ weights of a test chest x-ray image.

As shown in Fig. 1, the heatmap is computed for each test chest x-ray image to emphasize high-weight signals of COVID-19. The filters’ weights on the final convolutional layer are extracted to compute the final heatmap. Sample filters’ weights of one test chest x-ray image are shown in Fig. 3. In this example, the high-weights (i.e., yellow color) are located around lungs’ regions, because COVID-19 could damage lungs.

**Fig. 4:**
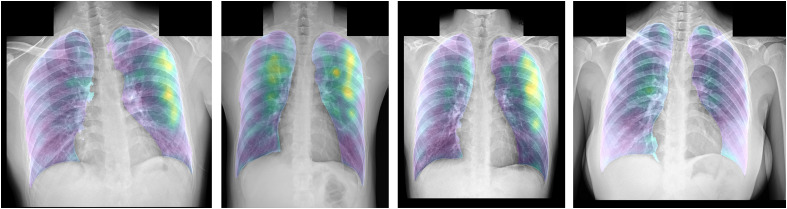
Sample final heatmaps of individual test chest x-ray images.

Then, the final heatmap is generated by averaging these filters’ weights. It is computed for individual test chest x-ray image. Sample final heatmaps are shown in Fig. 4. The first three heatmaps are computed from chest x-ray images with COVID-19. It can be seen that the high-weights of yellow patches are regions detected by the trained model to be signals of COVID-19. The medical experts can concentrate on these regions to final check the disease, while the final heatmap is computed from a chest x-ray image with the normal label. There are thus no yellow patches to represent any COVID-19 damage.

**Fig. 5:**
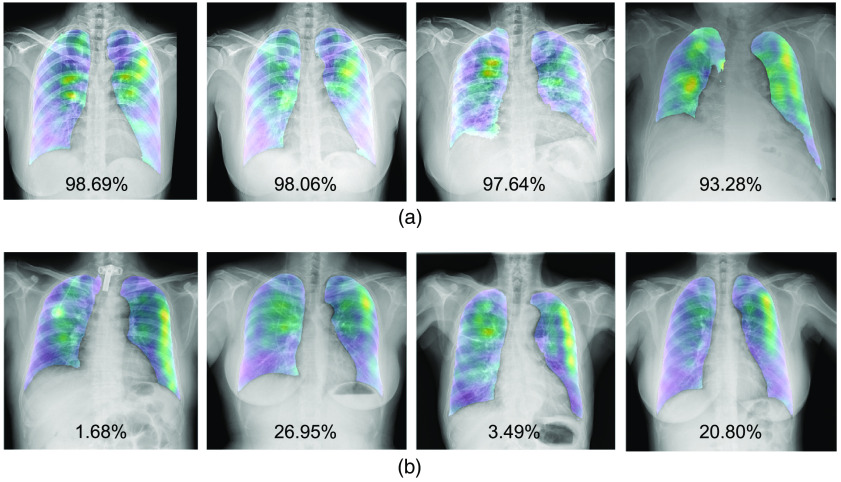
Sample final heatmaps with their confidence scores of being COVID-19. (a) Four examples of chest x-ray images with COVID-19 and (b) four examples of chest x-ray images with non-COVID-19.

Our solution also generates a confidence score of being COVID-19 for each test chest x-ray image. Four examples of chest x-ray images with COVID-19 and four examples of chest x-ray images with non-COVID-19 are shown in Fig. 5. It is clearly seen that the test images with COVID-19 could be classified correctly to be COVID-19 with very high confidence scores of above 90%. Also, the test images with non-COVID-19 could be classified correctly with very low confidence scores to be COVID-19 of below 30% or, in other words, with very high confidence scores to be non-COVID-19 of above 70%.

## 4 Discussion

The original trained model using the proposed method can classify a chest x-ray image into two classes of COVID-19 and non-COVID-19. The training and validating samples of the non-COVID-19 class are normal chest x-ray images without any remarks or diseases. This model is shown to achieve high performance on testing COVID-19 and normal chest x-ray images. This is mainly because the patterns of COVID-19 and normal cases are seen in the training process. However, its performance is significantly dropped when it is tested with chest x-ray images with other remarks or diseases such as fibrosis, spondylosis of spine, tuberculosis, pneumonia, and pulmonary edema.

This is as expected because patterns of other remarks or diseases are not seen and learned in the training process. Also, they occur in lungs’ regions as similar to COVID-19. So, they could be easily confused with COVID-19, using this developed model. It can result in many false detection/positive cases, which could not be acceptable for practical usages.

Therefore, the model is further improved by adding sample chest x-ray images containing other remarks and diseases in the training and validating processes. This makes the model to learn differences between patterns of COVID-19 and patterns of other diseases. It results in increasing the specificity score and reducing the false detection of COVID-19. However, the sensitivity score is also lower, when compared with the original trained model.

Then, the next version of the developed model is trained to classify a chest x-ray image into three classes of COVID-19, normal, and other diseases. This could maintain the sensitivity score and increase the specificity score. This is because separating the other diseases class from the non-COVID-19 class can reduce the confusion between COVID-19 and other diseases, and the confusion between normal and other diseases. As shown in Fig. 5, the COVID-19 cases are clearly separated from the non-COVID-19 cases (i.e., normal and other diseases), with very high confidence scores.

**Fig. 6:**
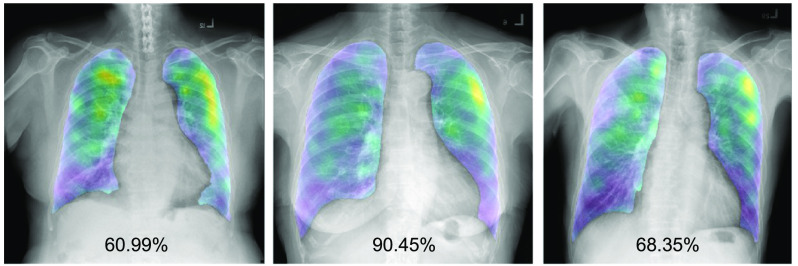
Sample final heatmaps with their confidence scores of the false-positive cases. The first two images are normal cases and the last image contains another abnormality.

As additionally reviewed by the expert, the generated heatmaps of the COVID-19 cases could identify areas of COVID-19 correctly. However, some heatmaps of the false-positive cases are reported incorrectly as shown in Fig. 6. The first two images are normal cases and the last image contains another abnormality. The confidence scores of being COVID-19 of the three images are all higher than 50%. However, to be classified as COVID-19 with the cut-off of 90%, only the second image is wrongly classified. In addition for these three cases, the heatmaps incorrectly highlight the non-COVID-19 cases as COVID-19 (i.e., yellow areas).

However, none of the developed models can achieve a perfect performance of 100% accuracy. Thus, they should be adopted for the prefiltering of normal cases, by cutting off chest x-ray images that are classified to be COVID-19 with very low scores—that is, they have high confidence to be non-COVID-19. In this way, it can be used to reduce a number of chest x-ray images that must be manually diagnosed by human experts.

In addition, the heatmap is generated to emphasize possible areas of being COVID-19 in each chest x-ray image. This can be an assistive tool for human experts to be used together with the computed confidence score, to conclude the final diagnosis.

## 5 Conclusions

This paper presents a solution for COVID-19 classification in chest x-ray images. Its backbone CNN architecture is developed using ResNet-101. The model is trained from scratch with a large size of the network’s input of 1500×1500  pixels. Data augmentation is also applied on the original training images to enhance the regularization of the model. It is developed in two versions of classification: two-classes-based and three-classes-based. The two-classes-based version is used to classify chest x-ray images into COVID-19 and non-COVID-19. The three-classes-based version is used to classify chest x-ray images into COVID-19, normal, and other abnormal. The proposed solution achieves very promising sensitivity, specificity, and accuracy of 97%, 98%, and 98%, respectively. The developed solution can also generate the heatmap with a confidence score of being COVID-19, to emphasize the result on each test image. The heatmap is visualized on only lung regions segmented using U-Net.
